# Data on structure and farming practices of French organic vegetable farms, with focus on the use of inputs and the socio-economic context

**DOI:** 10.1016/j.dib.2021.107184

**Published:** 2021-05-30

**Authors:** Antonin Pépin, Kevin Morel, Hayo M.G. van der Werf

**Affiliations:** a CTIFL^1^ Ctr St Remy, Route Mollèges, 13210 St Remy de Provence, France; b UMR SAS, INRAE^2^, Institut Agro, 35000 Rennes, France; c UMR SADAPT, INRAE, AgroParisTech, Université Paris-Saclay, 75005 Paris, France

**Keywords:** Organic farming, Vegetable farms, Farming diversity, Farming practice, Agroecology, Multivariate analysis

## Abstract

Organic vegetable farming systems in France have diverse farm structures, farming practices and socio-economic contexts. From April-July 2019, Pépin et al. [1] surveyed 165 farms using an online form. The questions about farming practices or socio-economic context did not require quantitative responses to make them simple and easy to answer. From a list of practices, farmers were asked which one(s) they used most often. Using decision rules, the answers were transformed into variables that are suitable for multivariate analysis. The data set also contains analysed data, including composite indexes derived from survey answers, as well as the number of the cluster to which each farm belonged, created after multivariate analysis and clustering performed on the data set.

**Specifications Table**

**Table:** 

Subject	Agricultural Sciences
Specific subject area	Organic vegetable production systems, Agroecology
Type of data	Table
How data were acquired	Online survey
Data format	Raw and analysed data
Parameters for data collection	The survey included only farms that produced organic vegetables for the fresh market as their main production. We focussed our survey on two contrasting regions of France: the north-west (Brittany, Normandy, Pays de la Loire) and south-east (Provence-Alpes-Côte-d'Azur, Languedoc-Roussillon). Because of word-of-mouth communication, however, some farmers in other regions answered the survey.
Description of data collection	Data were collected using an online survey, made with Google Forms, sent to farmers from April-July 2019. The online survey was disseminated to the farmers through several networks − specialised in organic farming or not − including local agricultural development organisations and commercial organisations, to capture as many farm types as possible. Follow-up e-mails and phone calls were made regularly based on the responses collected, to ensure that sampling was as complete as possible.
Data source location	Country: France
Data accessibility	Raw and analysed data are deposited in a public repository Repository name: INRAE dataverse (https://data.inrae.fr/) Data identification number: 10.15454/YAXXYH Direct URL to data: https://doi.org/10.15454/YAXXYH
Related research article	A. Pépin, K. Morel, H.M.G. van der Werf, Conventionalised vs. agroecological practices on organic vegetable farms: investigating the influence of farm structure in a bifurcation perspective, Agricultural Systems 190, 103129. https://doi.org/10.1016/j.agsy.2021.103129

**Value of the Data**

• The data set contains information on farming practices, with focus on the use of inputs and socio-economic issues in organic vegetable production, as well as farm and farmer characteristics.
• The data set can be used by other researchers who work on organic practices in vegetable farming from an agro-ecological perspective.
• These data can be used for future research on the relation between farm structure, farming practices in organic vegetable production and socio-economic elements.
• This data set includes data from 165 farms which represent a diverse sample.

## 1 Data Description

The data reported in this data paper derive from a survey of farm structure, farming practices and socio-economic context conducted in France based on 165 organic vegetable farms. The data set is composed of 1 text document, 3 PDF documents and 6 Excel files that contain raw or analysed data (Table 1). It is hosted on INRAE repository and it includes the survey form, the answers as raw data and analysed data. Pépin et al. [1] provide details about the method used to analyse data. Answers that were open-ended or contained personal data (e.g. name, e-mail, phone number) were excluded from the answer files.

**Table 1:** Contents of the data set.

File name	Description
*A0_README_description_of_files.txt*	Description of the files provided in the archive
*A1_Codebook_Variable_information.csv*	Table that presents the variables and their short names, full names, type (quantitative or categorical), units, possible values and details
*A2_Codebook_Categorical_Variable_values.csv*	Table that provides details about and explains the possible values taken by categorical values
*B1_Survey_form_Fr.pdf*	Original survey form created with Google Forms (in French)
*B2_Survey_form_Eng.pdf*	Survey form created with Google Forms, translated to English
*C1_Survey_answers_Fr.csv*	Original survey answers (in French)
*C2_Survey_answers_Eng.csv*	Survey answers, translated to English
*D_Decision_rules.pdf*	Document explaining how the survey answers were transformed into variables in the data set
*E_Dataset.csv*	Data set created based on the survey answers
*F_Composite_indexes.csv*	Data set with the values of the composite indexes calculated according to Pépin et al. [1]

**Fig. 1:**
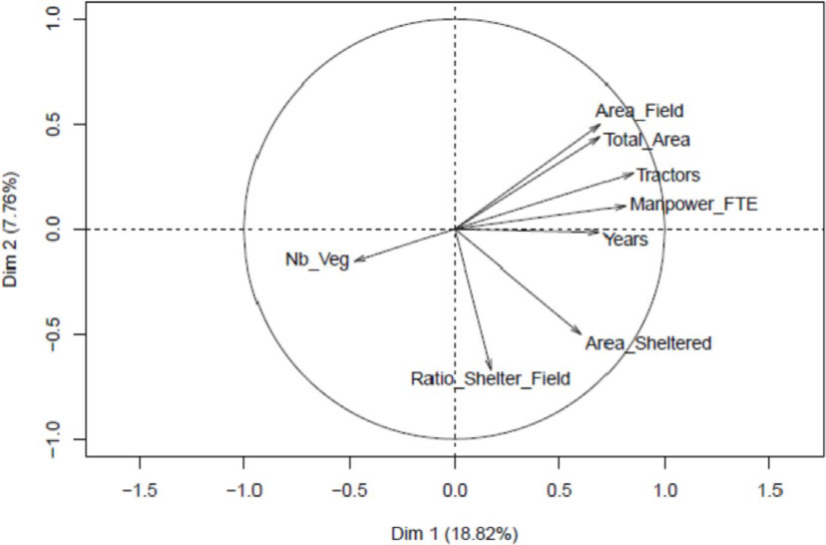
Correlation circle for the quantitative variables on the first two principal components.

**Fig. 2:**
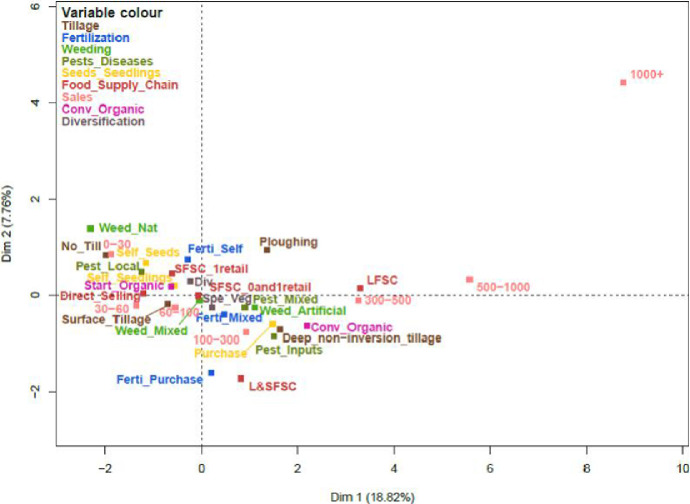
Representation of the categories of the categorical variables on the first two principal components.

**Fig. 3:**
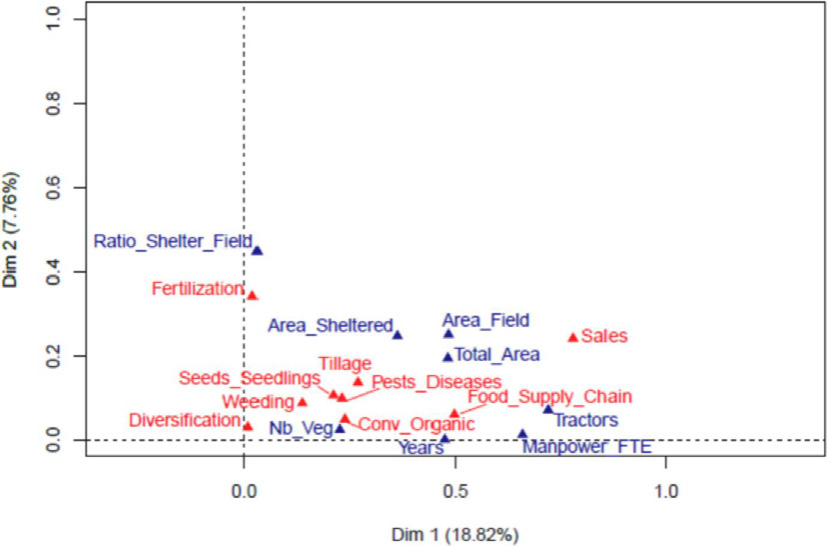
Associations between quantitative (blue) and categorical (red) variables on the first two principal components.

The results of the multivariate analysis conducted on the data set are presented in Table 2 and Fig. 1, Fig. 2, Fig. 3. It includes coordinates of the variables on the six principal components retained for the clustering, the correlation circle for the quantitative variables and the representation of the categories of the categorical variables on the first two principal components, and the associations between quantitative and categorical variables.

**Table 2:** Coordinates of the variables on the six principal components.

Variable	Dim.1	Dim.2	Dim.3	Dim.4	Dim.5	Dim.6
Years	0,4744189560	0,0002729537	0,0000089979	0,0055371181	0,0171702803	0,0484797620
Total_Area	0,4816120530	0,1951894910	0,0212584800	0,0000214685	0,0064296329	0,0016749360
Area_Field	0,4832810650	0,2516595948	0,0019664960	0,0025306635	0,0084659622	0,0266581040
Area_Sheltered	0,3640788340	0,2482546602	0,0856935000	0,0606381536	0,0008848295	0,0012093140
Manpower_FTE	0,6603791330	0,0120947450	0,0000826780	0,0615448804	0,0071853753	0,0155653500
Tractors	0,7219243690	0,0714604090	0,0204398900	0,0178606686	0,0026845719	0,0094871850
Ratio_Shelter_Field	0,0297155540	0,4469441867	0,1289005000	0,0187018880	0,0119505998	0,0008926270
Nb_Veg	0,2262670220	0,0241374533	0,2128739000	0,0108485159	0,0117312376	0,0205301750
Diversification	0,0090462030	0,0299649219	0,0009783325	0,0170704049	0,0030599415	0,2846180570
Fertilization	0,0191671450	0,3431179387	0,0283827600	0,0193806616	0,1888816132	0,0261087680
Tillage	0,2696908290	0,1371368889	0,4099002000	0,0512841738	0,1201344743	0,1305419100
Weeding	0,1364180930	0,0874620224	0,3041534000	0,3033157781	0,0692559995	0,0702891130
Pests_Diseases	0,2313476070	0,0991839132	0,0870709400	0,2884497764	0,1037189664	0,0771766340
Seeds_Seedlings	0,2111297320	0,1064088337	0,0780870600	0,0868591718	0,2238424723	0,1048908640
Food_Supply_Chain	0,4973274600	0,0604273794	0,2077630000	0,1879828808	0,3752937908	0,1597672070
Sales	0,7813459380	0,2424041787	0,3375564000	0,4108268194	0,2803084414	0,3520761180
Conv_Organic	0,2384361720	0,0490676290	0,0403923500	0,0017559832	0,0184547693	0,0409591350

## 2 Experimental Design, Materials and Methods

Data were collected using an online survey sent to farmers from April-July 2019. The survey was carried out in two French regions with contrasting types of vegetable production: the north-west (Brittany, Normandy, Pays de la Loire) and south-east (Provence-Alpes-Côte-d'Azur, Languedoc-Roussillon).

The survey targeted farms that produced organic vegetables for the fresh market as their main production. The online survey, designed using Google Forms, was disseminated to farmers through several networks, including local agricultural development organisations and commercial organisations. Farmers completed 174 surveys, 165 of which were sufficiently complete to be included in the data set. In particular, we excluded six farms created in 2019 (i.e. less than one year of experience) and three farms that were not professional farms. Most farmers who answered the survey were located in the two targeted regions, but because of word-of-mouth communication, some farmers in other regions answered it.

The survey's questions focussed on farm structure, farming practices and socio-economic issues. The questions were divided into six categories:

- Farm history and geography
  ○ Farm age
  ○ Years since the farm was labelled “organic”
  ○ Location (administrative department)
- Land
  ○ Utilised agricultural area including non-cultivated areas
  ○ Area cultivated in vegetables, whether outdoors or sheltered (high plastic tunnels or multi-span greenhouses)
- Human and mechanical labour resources
  ○ Number of people working permanently or temporarily (labour)
  ○ Number of tractors
- Production
  ○ Number of different vegetables grown: farmers were asked to count the types of vegetables distinguished by consumers and marketing, regardless of their botanical species [2]. For example, cauliflower and kale are two different vegetables, as are green beans and dried beans. No distinction was made between varieties. Lettuce (e.g. Batavia, oakleaf) counted as one vegetable type. Potatoes and strawberries were considered vegetables.
  ○ Other types of production besides vegetables
- Farming practices
  ○ Type of tillage and tools used
  ○ Main practices to manage soil fertility
  ○ Main practices to control weeds
  ○ Main practices to control pests and diseases
  ○ Actions to protect or promote local biodiversity
  ○ Origin of seeds and seedlings
- Economy and selling strategy
  ○ Marketing supply chains
  ○ Destination of the vegetables sold (from local to export markets)
  ○ Annual revenue

As detailed online surveys that take too much time to fill out may deter the people targeted, the questions about farming practices or socio-economic context did not require quantitative responses, in order to make them easier to answer. In most cases, farmers were asked multiple-choice questions about which practices they used most often.

The answers were transformed into variables according to decision rules, as explained in *Decision_rules.pdf*. Missing answers were imputed using regularised iterative algorithms [1]. The variables and imputed values are available in *Dataset.csv*. The variables are suitable for statistical analyses such as multivariate analyses.

A subset of variables was transformed into normalised primary indicators. An additive combination of these indicators was calculated, which yielded composite indexes [1]. The values of these indicators and indexes for each farm are shown in *Composite_indexes.csv*.

Based on the data set, a farm typology was developed using Factor Analysis of Mixed Data and agglomerative hierarchical clustering (AHC) [1]. The resulting farm clusters are shown in *Composite_indexes.csv* and described by Pépin et al. [1]. Table 2 provides coordinates of the variables on the six principal components retained for the AHC.

Figs. 1 and 2 respectively represent the correlation circle for the quantitative variables and the categories of the categorical variables on the first two principal components. Fig. 3 represents the associations between quantitative and categorical variables.

## Ethics Statement

All data were analysed anonymously. The farmers participated in the survey voluntarily and have agreed in writing to publication of the anonymised survey data for research purposes.

## CRediT Author Statement

**Antonin Pépin:** Conceptualization, Methodology, Software, Writing - original draft preparation; **Kevin Morel**: Conceptualization, Writing - reviewing & editing; **Hayo van der Werf:** Conceptualization, Writing - reviewing & editing.

## Declaration of Competing Interest

The authors declare that they have no known competing financial interests or personal relationships which have, or could be perceived to have, influenced the work reported in this article.

## Appendix Supplementary materials

Image, application 1

Image, application 2

Image, application 3

Image, application 4

Image, application 5

Image, application 6

Image, application 7

Image, application 8

Image, application 9

Image, application 10
