# Supplementary material for: Data on structure and farming practices of French organic vegetable farms, with focus on the use of inputs and the socio-economic context
Source: Data Brief. 2021 May 30;37:107184. doi: 10.1016/j.dib.2021.107184 (PMC8207183; doi:10.1016/j.dib.2021.107184)
Supplement: Supplementary file 4 [file mmc4.pdf]

Study on the environmental performance of organic vegetable farms

*Required

Characteristics of the farm

1. Year of establishment : *

____

2. Year of conversion to organic farming : *

____

3. In which administrative department is your farm located? (department number) *

____

4. What certifications or specifications do you have, including private or regional brands?

Tick all that apply.

- [ ] Organic farming certification (Ecocert, ...)
- [ ] Biocohérence
- [ ] Nature et Progrès
- [ ] Biodynamie (Demeter...)
- [ ] Global Gap
- [ ] ISO 14001

Other: [ ] _______________

5. What is the Useful Agricultural Area (UAA) of your farm? (in hectares) *

6. What is the area cultivated with vegetables? (including footpaths) *

7. What outdoor vegetable area do you cultivate? (in hectares) *

8. What area do you cultivate under unheated shelter? (in hectares or m^2) *

9. What area do you cultivate in heated greenhouses? (in hectares or m^2) *

10. What other type of production do you have, if any? (other than vegetables) *

11. Are all your surfaces organic? *

Mark only one oval.

- Yes *Skip to question 13*
- No *Skip to question 12*

Surfaces in Organic Farming

12. What area for organic vegetables? *

Labour

13. How many family workers or associates (self-employed) work on the farm? *

(in full-time equivalent)

14. How many permanent workers do you employ? *

(in full-time equivalent)

15. How many seasonal workers do you employ at the peak of work? *

(a seasonal worker counts as 1, regardless of the duration of his work)

16. How many tractors do you own? *

Production methods and cultural practices

17. How many different vegetables do you produce? (if there are many, an order of magnitude is sufficient) *

We count here the types of vegetables in the "general public" sense. For example, cauliflower and green cabbage are 2 different vegetables; the same goes for green beans and dry beans. There is no distinction between varieties: for example yellow onion and red onion count for 1. Salads (lettuce, batavia, etc.) count for 1.

18. What are the main vegetables? *

if they are very diverse, indicate "diverse"

19. Which of the following fertilisers do you mainly use? As a secondary fertiliser? Never ? *

Mark only one oval per row.

	Main	Secondary	Never
Self- or locally produced farm fertiliser (animal origin) (manure, slurry, etc.)	⬭	⬭	⬭
Organic commercial fertilizer	⬭	⬭	⬭
Self-produced compost	⬭	⬭	⬭
Purchased compost	⬭	⬭	⬭
Green manure	⬭	⬭	⬭

20. If you want to provide more details (brand of fertilizer, type of manure, tonnages ...), or if the list of fertilizers does not correspond to you, it's here!

21. What type of tillage do you use? *

Mark only one oval per row.

	Nearly all of the area (>75%)	Moderate part of the area (25-75%)	Small part of the area (<25%)	Never
Ploughing (tillage with turning)				
Deep non-inversion tillage				
Surface tillage				
No-tillage				

22. The tillage is essentially:

Mark only one oval per row.

	Tractor	Garden cultivator	Animal traction	Manual	Not concerned
Open field					
Under shelter					

23. If you want to clarify the tillage, or if the proposals do not suit you, it's here!

24. Which of the following weed control strategies do you mainly use? As a secondary strategy? Never? *

Mark only one oval per row.

	Main	Secondary	Never
Plastic mulching	⬭	⬭	⬭
Woven plastic mulching	⬭	⬭	⬭
Biodegradable plastic mulching	⬭	⬭	⬭
Occultation (tarpaulin) between two crops	⬭	⬭	⬭
Plant mulch	⬭	⬭	⬭
Manual weeding (hoeing, etc.)	⬭	⬭	⬭
Mechanical weeding (spring tine harrow, towed hoe, etc.)	⬭	⬭	⬭
False sowing, tillage	⬭	⬭	⬭
Steam or flaming	⬭	⬭	⬭
Solarization (increasing temperature by covering the ground with transparent plastic film)	⬭	⬭	⬭

25. If you want to provide more details on weeding, or if the proposals do not suit you, it's here!

____

____

____

____

____

26. Which of the following pest and disease control strategies do you mainly use? As a secondary strategy? Never? *

Mark only one oval per row.

	Main	Secondary	Never
Copper			
Sulfur			
Biocontrol products (Bt, pheromones, micro-organisms, etc.)			
Releases of pest predators			
Rely on the surrounding biodiversity			
Association of crops			
Service plants (repellents, traps, etc.)			
Home-made products (plant slurry, plant decoctions, etc.)			

27. If you want to provide more details on the method of struggle, or if the proposals do not suit you, it's here!

28. Some producers voluntarily leave or maintain spaces in order to promote biodiversity (hedges, fallows, extensive meadows, ponds, flowering plants, etc.). On your farm, this approach is: *

Mark only one oval.

- Central: it lies at the heart of the production system
- Important: you devote time and space
- Unimportant: you think about it without spending a lot of time or space
- Minor

29. If you want to provide more details on spaces for biodiversity, it's here!

____
____
____
____
____

30. Seeds and seedling management

Mark only one oval.

- You produce your own seeds and seedlings (at least in part)
- You produce your own seedlings (at least in part) from purchased seeds
- You buy your seeds and seedlings produced locally
- ou buy your seeds and seedlings from a large company

31. If you want to clarify the seeds and seedlings, it's here!

____
____
____
____
____

32. What cultivation practices or methods correspond to your farm? *

Mark only one oval per row.

	Matches well	Match relatively well	Does not match
One crop per plot or per tunnel			
Several crops on the same plot or in the same tunnel			
Intercropping (search for interactions)			
"Alternative" agriculture (Permaculture and other trends)			
Agroforestry or market gardening orchard			
Market gardening on Living Soil ("MSV" in French)			

33. If you want to clarify your farming methods, it's here!

Socio-economic context

34. Do you use these marketing channels mainly? As a secondary channel? Never? *

Mark only one oval per row.

	Main	Secondary	Never
Direct selling (market, baskets, etc.)	⬭	⬭	⬭
Other short food supply chain (1 intermediary)	⬭	⬭	⬭
Wholesaler or shipper	⬭	⬭	⬭
Other long food supply chain (2+ intermediaries)	⬭	⬭	⬭
Cooperative	⬭	⬭	⬭

35. Your products are marketed: *

Tick all that apply.

- [ ] In the departement
- [ ] In the region
- [ ] In France
- [ ] Abroad

36. If you want to provide more details, or if the proposals do not suit you, it's here!

____
____
____
____
____

37. What is the turnover (revenue) of your farm? *

Mark only one oval.

- <30 000€
- 30 000 - 60 000 €
- 60 000 - 100 000 €
- 100 000 - 300 000 €
- 300 000 - 500 000 €
- 500 000 - 1 000 000 €
- > 1 000 000 €

Contact

38. Farm name

____

39. Your name / first name

____

40. Are you interested in the results of the study? (if yes, remember to indicate your e-mail below) *

Mark only one oval.

- Yes
- No

41. Your e-mail

____

42. Your phone number

43. Do you authorize me to contact you again for the rest of the study? (without any commitment) *

Mark only one oval.

- Yes
- No

You can help me even more by sending the questionnaire to other organic producers, word of mouth is always the most effective.

Thank you very much for your answers!

Don't forget to click on "send" below!

This content is neither created nor endorsed by Google.

Google Forms
