# Supplementary material for: Data on structure and farming practices of French organic vegetable farms, with focus on the use of inputs and the socio-economic context
Source: Data Brief. 2021 May 30;37:107184. doi: 10.1016/j.dib.2021.107184 (PMC8207183; doi:10.1016/j.dib.2021.107184)
Supplement: Supplementary file 7 [file mmc7.pdf]

Decision rules used to transform survey answers into variables

This document lists the variables created for survey answers. The full name of each variable is followed by its short name (in brackets).

Utilised agricultural area (UAA) (ha) (Total_Area)

The variable “Utilised agricultural area (UAA) (ha)” was created using the answer to the question:

- What is the Useful Agricultural Area (UAA) of your farm? (in hectares)

Outdoor vegetable area (ha) (Area_Field)

The variable “Outdoor vegetable area (ha)” was created using the answer to the question:

- What outdoor vegetable area do you cultivate? (in hectares)

Sheltered vegetable area (ha) (Area_Sheltered)

The variable “Sheltered vegetable area (ha)” was created using the answer to the question:

- What area do you cultivate under unheated shelter? (in hectares or m^2)

Sheltered area as % of vegetable area (Ratio_Shelter_Field)

The variable “Sheltered area as % of vegetable area” was created using this equation:

- Ratio_Shelter_Field = Area_Sheltered / (Area_Sheltered + Area_Field)

Labour (full-time equivalent) (Manpower_FTE)

The variable “Labour (full-time equivalent)” was created using the answer to these questions:

- How many family workers or associates (self-employed) work on the farm?
- How many permanent workers do you employ?
- How many seasonal workers do you employ at the peak of work?

We assumed that the two first categories worked full time and the seasonal workers were employed 4 months per year.

Number of tractors (Tractors)

The variable "Number of tractors" was created using the answer to the question:

- How many tractors do you own?

Number of types of vegetables (Nb_Veg)

The variable "Number of types of vegetables" was created using the answer to the question:

- How many different vegetables do you produce?

Tillage (Tillage)

The variable "Tillage" was created using the answer to the question:

- What type of tillage do you use, and is it on nearly all of the area (>75%), a moderate part of the area (25-75%), or a small part of the area (<25%)?

according to the following rules:

Short name	Long name	Decision rule
**No_Till**	No-tillage	[No-tillage] is applied to the largest share of cultivated area, and all other tillage types are applied to a smaller share of cultivated area
**Surface_Tillage**	Surface tillage	[Surface tillage] is applied to the largest share of cultivated area **AND** to an area larger than [Deep non-inversion tillage] **AND** to an area larger than [Ploughing]
**Deep_non-inversion_tillage**	Deep non-inversion tillage	[Deep non-inversion tillage] is applied to the largest share of cultivated area **AND** to an area larger than [Ploughing]
**Ploughing**	Ploughing	[Ploughing] is applied to the largest share of cultivated area

Fertilisation (Fertilization)

The variable "Fertilisation" was created using the answer to the question:

- Which of the following fertilisers do you mainly use? As a secondary fertiliser? Never?

according to the following rules:

Short name	Long name	Decision rule
**Ferti_Self**	Self- or locally produced	[Self- or locally produced farm fertiliser (animal origin)] **OR** [Self-produced compost] **OU** [Green manure] = Main<br>**AND**<br>[Organic commercial fertilizer] **AND** [Purchased compost] = Secondary or Never
**Ferti_Mixed**	Mixed	At least one "Main" for:<br>- [Self- or locally produced farm fertiliser (animal origin)] **OR** [Self-produced compost] **OR** [Green manure]<br>**AND**<br>- [Organic commercial fertilizer] **OR** [Purchased compost]
**Ferti_Purchase**	Purchased on the market	[Organic commercial fertilizer] **OR** [Purchased compost] = Main<br>**AND**<br>[Self- or locally produced farm fertiliser (animal origin)] **AND** [Self-produced compost] **AND** [Green manure] = Secondary or Never

Weed control (Weeding)

The variable "Weed control" was created using the answer to the question:

- Which of the following weed control strategies do you mainly use? As a secondary strategy? Never?

according to the following rules:

Short name	Long name	Decision rule
**Weed_Nat**	Based on natural techniques	[Plant mulch] **OR** [Manual weeding] **OR** [Mechanical weeding] **OR** [False sowing, tillage] = Main<br>**AND** other categories = Secondary or Never
**Weed_Mixed**	Mixed	Other combinations
**Weed_Artificial**	Based on artificialising the environment	[Plastic mulching] **OR** [Woven plastic mulching] **OR** [Biodegradable plastic mulching] **OR** [Occultation (tarpaulin) between two crops] **OR** [Steam or flaming] **OR** [Solarization] = Main<br>**AND** other categories = Secondary or Never

Pest and disease control (Pests_Diseases)

The variable "Pest and disease control" was created using the answer to the question:

- Which of the following pest and disease control strategies do you mainly use? As a secondary strategy? Never?

according to the following rules:

Short name	Long name	Decision rule
**Pest_Local**	Based on local resources	[Rely on the surrounding biodiversity] **OR** [Association of crops] **OR** [Service plants] **OR** [Home-made products] = Main **AND** other categories = Secondary or Never
**Pest_Mixed**	Mixed	Other combinations
**Pest_Inputs**	Based on external inputs	[Copper] **OR** [Sulfur] **OR** [Biocontrol products] **OR** [Releases of pest predators] = Main **AND** other categories = Secondary or Never

Seed and seedling management (Seeds_Seedlings)

The variable "Seed and seedling management" was created using the answer to the item:

- Seeds and seedling management

according to the following rules:

Short name	Long name	Decision rule
**Self_Seeds**	Seeds partly self-produced	You produce your own seeds and seedlings (at least in part)
**Self_Seedlings**	Seedlings partly self-produced	You produce your own seedlings (at least in part) from purchased seeds
**Purchase**	Seeds and seedlings purchased	You buy your seeds and seedlings produced locally **OR** You buy your seeds and seedlings from a large company

Willingness to dedicate space to biodiversity on the farm (Biodiversity)

The variable "Willingness to dedicate space to biodiversity on the farm" was created using the answer to the item:

- Some producers voluntarily leave or maintain spaces in order to promote biodiversity (hedges, fallows, extensive meadows, ponds, flowering plants, etc.). On your farm, this approach is:

according to the following rule:

Short name	Long name	Decision rule
**Major**	Major	Central: it lies at the heart of the production system
**Important**	Important	Important: you devote time and space
**Unimportant**	Unimportant	Unimportant: you think about it without spending a lot of time or space **OR** Minor

Food supply chain (Food_Supply_Chain)

The variable "Food supply chain" was created using the answer to the question:

- Do you use these marketing channels mainly? As a secondary channel? Never?

according to the following rules:

Short name	Long name	Decision rule
**Direct_Selling**	Direct Selling (0 intermediary)	[Direct selling] = Main<br>**AND** other categories are not Main
**SFSC_0and1retail**	Direct selling and short mixed	[Direct selling] = Main<br>**AND** [Other short food supply chain (1 intermediary)] = Main<br>**AND** other categories are not Main
**SFSC_1retail**	Short (1 intermediary)	[Other short food supply chain (1 intermediary)] = Main<br>**AND** other categories are not Main
**L&SFSC**	Long and short mixed	[Wholesaler or shipper] **OR** [Other long food supply chain (2+ intermediaries)] **OR** [Cooperative] = Main<br>**AND** [Direct selling] **OR** [Direct selling] = Main
**LFSC**	Long (2+ intermediaries)	[Wholesaler or shipper] **OR** [Other long food supply chain (2+ intermediaries)] **OR** [Cooperative] = Main<br>**AND** other categories are not Main

Furthest selling destination (Selling_Destination)

The variable "Furthest selling destination" was created using the answer to the question:

- Your products are marketed: In the department? In the region? In France? Abroad?

according to the following rules:

Short name	Long name	Decision rule
**3_Department_Selling**	Department	"In the department" is the furthest selling destination
**2_Regional_Selling**	Regional	"In the region" is the furthest selling destination
**1_National_Selling**	National	"In France" is the furthest selling destination
**0_Foreign_Selling**	Foreign	"Abroad" is the furthest selling destination

Annual revenue in thousand Euros (Sales)

The variable "Annual revenue in thousand Euros" was created using the answer to the question:

- What is the turnover (revenue) of your farm?

according to the following rules:

Short name	Long name	Decision rule
**0-30**	0-30	< 30 000 €
**30-60**	30-60	30 000 - 60 000 €
**60-100**	60-100	60 000 - 100 000 €
**100-300**	100-300	100 000 - 300 000 €
**300-500**	300-500	300 000 - 500 000 €
**500-1000**	500-1000	500 000 - 1 000 000 €
**1000+**	1000+	> 1 000 000 €

Region (Region)

The variable “Region” was created using the answer to the question:

- In which administrative department is your farm located? (department number)

according to the following rules:

Short name	Long name	Decision rule
**SE**	South-east	Departments no. 04, 05, 06, 13, 83, 84, 11,30, 34, 48, 66
**NW**	North-west	Departments no. 22, 29, 35, 56, 44, 49, 53, 72, 85, 14, 50, 61
**SW**	South-west	Departments no. 24, 33, 40, 47, 64, 09, 12, 31, 32, 46, 65, 81, 82
**Other**	Other	Any other department

Conversion to organic farming (Conv_Organic)

The variable “Conversion to organic farming” was created using the answer to the items:

- Year of establishment:
- Year of conversion to organic farming:

according to the following rules:

Short name	Long name	Decision rule
**Start_Organic**	Farm created as organic	Year of conversion is not later than that of establishment
**Conv_Organic**	Farm converted to organic farming	Year of conversion is later than that of establishment

Diversification (Diversification)

The variable “Diversification” was created using the answer to the item:

- Diversification with other type of production (other than vegetables)

according to the following rules:

Short name	Long name	Decision rule
**Specialised**	Specialised in vegetable production	Specialised
**Diversified**	Diversified in other types of production	Diversified

Alternative farming (Alter_Farming)

The variable "Alternative farming" was created using the answer to the question:

- What cultivation practices or methods correspond to your farm?

according to the following rules:

Short name	Long name	Decision rule
**Alter**	Alternative farming	[Intercropping (search for interactions)] **OR** [Alternative agriculture (Permaculture and other trends)] **OR** [Agroforestry or market gardening orchard] **OR** [Market gardening on Living Soil ("MSV" in French)] = Matches well<br>**OR**<br>[Alternative agriculture (Permaculture and other trends)] = Matches relatively well
**Non_alter**	Non-alternative farming	Other combinations
