# Supplementary material for: Data on structure and farming practices of French organic vegetable farms, with focus on the use of inputs and the socio-economic context
Source: Data Brief. 2021 May 30;37:107184. doi: 10.1016/j.dib.2021.107184 (PMC8207183; doi:10.1016/j.dib.2021.107184)
Supplement: Supplementary file 10 [file mmc10.pdf]

Étude sur les performances environnementales des fermes maraichères et légumières bio

*Obligatoire

Caractéristiques de la ferme

1. Année d'installation : *

2. Année de conversion bio : *

3. Dans quel département se trouve votre ferme ? (n°) *

4. Quelles certifications ou cahier des charges avez-vous, y compris marques privées ou régionales ?

Plusieurs réponses possibles.

- [ ] Certification AB (Ecocert, ...)
- [ ] Biocohérence
- [ ] Nature et Progrès
- [ ] Biodynamie (Demeter...)
- [ ] Global Gap
- [ ] ISO 14001

Autre : [ ] ____________________

5. Quelle est la Surface Agricole Utile (SAU) de votre ferme ? (en hectare) *

6. Quelle est la surface cultivée en légumes ? (passe-pieds inclus) *

7. Quelle surface de légumes cultivez-vous en plein champ ? (en hectare) *

8. Quelle surface cultivez-vous sous abri froid ? (en hectare ou m²) *

9. Quelle surface cultivez-vous en serres chauffées ? (en hectare ou m²) *

10. Quelles autres productions avez-vous, si vous en avez ? (autre que légumes) *

11. Toutes vos surfaces sont-elles en bio ? *

Une seule réponse possible.

- Oui *Passer à la question 13*
- Non *Passer à la question 12*

Surfaces en bio

12. Quelle surface en légumes bio ? *

Le travail

13. Combien êtes-vous de travailleurs familiaux ou associés (non salariés) ? *

(en équivalent temps plein)

14. Combien employez-vous de salariés permanents ? *

(en équivalent temps plein)

15. Combien employez-vous de salariés saisonniers au maximum du pic de travail ? *

(un saisonnier compte pour 1, peu importe la durée de son travail)

16. Combien de tracteurs possédez-vous ? *

Modes de production et pratiques culturales

17. Combien de légumes différents produisez-vous ? (s'il y en a beaucoup, un ordre de grandeur suffit) *

On compte ici les types de légumes au sens "grand public". Par exemple, chou-fleur et chou vert sont 2 légumes différents; idem pour haricot vert et haricots secs. On ne distingue pas les variétés : par exemple oignon jaune et oignon rouge comptent pour 1. Les salades (laitue, batavia, etc.) comptent pour 1.

18. Quels sont les principaux légumes ? *

si vous êtes très diversifié, indiquez "diversifié"

19. Parmi les engrais suivants, lesquels utilisez-vous de façon principale ? secondaire ? jamais ? *

Une seule réponse possible par ligne.

	Principale	Secondaire	Jamais
Engrais de ferme (origine animale) auto-produit ou produit localement (fumier, lisier...)			
Engrais organique du commerce			
Compost auto-produit			
Compost du commerce			
Engrais verts			

20. Si vous voulez apporter des précisions (marque de l'engrais, type de fumier, tonnages...), ou si la liste d'engrais ne vous correspond pas, c'est ici !

21. Quel type de travail du sol effectuez-vous ? *

Une seule réponse possible par ligne.

	Toutes les surfaces ou presque (entre 75% et 100%)	Une partie des surfaces (entre 25% et 75%)	Une faible partie des surfaces (moins de 25%)	Jamais
Labour (travail du sol avec retournement)				
Décompactage (travail profond, sans retournement)				
Travail du sol superficiel (outils à griffes)				
Pas de travail du sol				

22. Le travail du sol est essentiellement :

Une seule réponse possible par ligne.

	Tracteur	Motoculteur	Traction animale	Manuel	Non concerné
Plein champ					
Sous abri					

23. Si vous voulez apporter des précisions sur le travail du sol, ou si les propositions ne vous correspondent pas, c'est ici !

24. Parmi les stratégies de lutte contre les adventices suivantes, lesquelles utilisez-vous de façon principale ? secondaire ? jamais ? *

Une seule réponse possible par ligne.

	Principale	Secondaire	Jamais
Paillage plastique (bâche noire)			
Paillage bâche tissée			
Bâche biodégradable			
Occultation (bâche) entre 2 mises en culture			
Paillage végétal (mulch)			
Désherbage manuel (binage, sarclage...)			
Désherbage mécanique (herse étrille, bineuse tractée...)			
Faux semis, travail du sol			
Vapeur ou brulage			
Solarisation (élévation de la température en recouvrant le sol d'un film plastique transparent)			

25. Si vous voulez apporter des précisions sur le désherbage, ou si les propositions ne vous correspondent pas, c'est ici !

26. Parmi les stratégies suivantes de lutte contre les ravageurs et maladies, lesquelles utilisez-vous de façon principale ? secondaire ? jamais ? *

Une seule réponse possible par ligne.

	Principale	Secondaire	Jamais
Cuivre	⬭	⬭	⬭
Soufre	⬭	⬭	⬭
Produits de bio-contrôle (Bt, phéromones, micro-organismes...)	⬭	⬭	⬭
Lâchers de prédateurs des ravageurs	⬭	⬭	⬭
S'appuyer sur la biodiversité environnante	⬭	⬭	⬭
Association de cultures	⬭	⬭	⬭
Plantes de service (répulsion, plantes pièges...)	⬭	⬭	⬭
PNPP (purins, décoctions végétales...)	⬭	⬭	⬭

27. Si vous voulez apporter des précisions sur les méthode de lutte, ou si les propositions ne vous correspondent pas, c'est ici !

____

____

____

____

____

28. Certains producteurs laissent volontairement ou entretiennent des espaces dans le but de favoriser la biodiversité (haies, jachères, prairies extensives, mares, plantes à fleur, etc.). Dans votre ferme, cette démarche est : *

Une seule réponse possible.

- centrale, c'est au coeur du système de production
- importante, vous y consacrez du temps et de l'espace
- peu importante, vous y pensez sans y consacrer beaucoup de temps ni d'espace
- mineure

29. Si vous voulez apporter des précisions sur les espaces pour la biodiversité, c'est ici !

30. Gestion des semences et plants

Une seule réponse possible.

- Vous produisez vos propres semences et plants (au moins en partie)
- Vous produisez vos propres plants (au moins en partie) à partir de semences achetées
- Vous achetez vos semences et plants produits localement
- Vous achetez vos semences et plants à une grande entreprise

31. Si vous voulez apporter des précisions sur les semences et plants, c'est ici !

32. Quelles pratiques ou modes de culture correspondent à votre ferme ? *

Une seule réponse possible par ligne.

	Correspond bien	Correspond plutôt bien	Ne correspond pas
Une seule culture par parcelle ou par tunnel			
Plusieurs cultures sur une même parcelle ou dans un même tunnel			
Associations de cultures (recherche d'interactions)			
Agricultures "alternatives" (Permaculture et autres courants)			
Agroforesterie ou Verger maraîcher			
Maraichage sur Sol Vivant (MSV)			

33. Si vous voulez apporter des précisions sur vos modes de cultures, c'est ici !

Commercialisation

34. Utilisez-vous ces voies de commercialisation de façon : principale ? secondaire ? jamais utilisées ? *

Une seule réponse possible par ligne.

	Principale	Secondaire	Jamais
Vente directe (marché, paniers...)			
Autre circuits courts (1 intermédiaire)			
Grossiste ou expéditeur			
Autres circuits longs (au moins 2 intermédiaires)			
Coopérative			

35. Vos produits sont commercialisés : *

Plusieurs réponses possibles.

- [ ] Dans le département
- [ ] Dans la région
- [ ] En France
- [ ] à l'étranger

36. Si vous voulez apporter des précisions, ou si les propositions ne vous correspondent pas, c'est ici !

37. Quel est le chiffre d'affaires de votre ferme ? *

Une seule réponse possible.

- <30 000€
- entre 30 000 et 60 000 €
- entre 60 000 et 100 000 €
- entre 100 000 et 300 000 €
- entre 300 000 et 500 000 €
- entre 500 000 et 1 000 000 €
- > 1 000 000 €

Contact

38. Nom de la ferme

____

39. Votre nom / prénom

____

40. Êtes-vous intéressés par les résultats de l’étude ? (si oui, pensez à indiquer votre e-mail ci-dessous) *

Une seule réponse possible.

- Oui
- Non

41. Votre e-mail

____

42. Votre n° de téléphone

____

43. M’autorisez-vous à vous recontacter pour la suite de l’étude ? (sans aucun engagement) *

Une seule réponse possible.

- Oui
- Non

Vous pouvez m'aider encore plus en transmettant le questionnaire à d'autres producteurs bio, le bouche à oreilles est toujours le plus efficace.

Merci infiniment pour vos réponses !

N'oubliez pas de cliquer sur "envoyer" ci-dessous!

Ce contenu n'est ni rédigé, ni cautionné par Google.

Google Forms
